# Ready to participate? Using qualitative data to typify older adults’ reasons for (non-) participation in a physical activity promotion intervention

**DOI:** 10.1186/s12889-019-7688-y

**Published:** 2019-10-22

**Authors:** Frauke Wichmann, Tilman Brand, Dirk Gansefort, Ingrid Darmann-Finck

**Affiliations:** 1 Department 4: Qualification and Curriculum Research, Institute of Public Health and Nursing Research – IPP, University of Bremen, Grazer Straße 4, 28359 Bremen, Germany; 2 Department of Prevention and Evaluation, Leibniz Institute for Prevention Research and Epidemiology – BIPS, Bremen, Germany; 3 Landesvereinigung für Gesundheit und Akademie für Sozialmedizin Niedersachsen e.V, Hannover, Germany; 4 Health Sciences Bremen, University of Bremen, Bremen, Germany

**Keywords:** Physical activity, Older adults, Primary prevention, Physical activity interventions, Activity goals, Time management, Participation, Recruitment

## Abstract

**Background:**

A sufficient amount of regular moderate physical activity (PA), at least 2.5 h of moderate to vigorous PA per week as recommended by the WHO, is one of the most important lifestyle factors for maintaining good health in old age. However, less than one in four older adults (65 years or older) in Germany meets this recommendation for PA. Although previous research has found several factors related to participation in PA programmes, little is known about how these factors simultaneously affect participation decisions of older adults and how PA programmes can accommodate these dynamics. Taking an everyday life perspective, this study aimed to identify multidimensional types of PA behaviour among older adults.

**Methods:**

In this qualitative study, 25 episodic interviews were conducted with participants and non-participants (ratio 1:3) of a structured PA intervention for older adults (65 years or older). Direct and indirect recruitment methods (e.g. pick up, gatekeeper) were used in different municipalities in Northwest Germany. The interviews were analysed according to the Grounded Theory methodology and a typology of PA participation behaviour was derived from the responses of the interviewees.

**Results:**

Four types of PA participation behaviour were identified based on different activity goals and time management preferences: ‘Health designer’, ‘Flexible function-oriented type’, ‘Comparison and competition type’ and ‘Fun and wellness-oriented type’. The results indicate that the structured PA intervention was better able to reach the health designer and the competitive type but was less successful in accommodating the function- or wellness-oriented type.

**Conclusions:**

In order to improve older adults’ participation in PA, preventive offers should take various activity goals and the desire for flexible time management in everyday life into account. The typology of PA participation behaviour contributes to a better understanding of the target group and can thus help to improve the development, communication and implementation of tailored PA interventions.

## Background

About one fifth (21%) of the German population is 65 years or older and this population group is continuing to rise, as is being observed in other European countries [1, 2]. The WHO considers physical activity (PA) as one of the most important lifestyle factors supporting healthy ageing and recommends at least 2.5 h of moderate to vigorous PA per week. The same recommendation can be found in the Global Plan of Action for the Prevention and Control of Noncommunicable Diseases 2013–2020 [3–5]. However, only less than a fourth of the older population in Germany meets this recommendation [6]. Strategies to increase the reach, acceptance and effectiveness of PA interventions in older adults are therefore of high public health relevance [7–9]. Although structured PA interventions may increase the level of PA in older age [10], not all persons in the age group (≥ 65 years) are equally reached by PA offers [9–11]. Regarding participation in individual-oriented health promotion programmes, differences across social strata and between men and women have been shown by previous research [12]. Further, various factors at different social-ecological levels have been shown to affect participation in health promotion programmes [13, 14]. It has been suggested that older adults’ participation in PA interventions is influenced by system-level factors, such as availability and accessibility of PA programmes and social support [13, 15–17], as well as by intervention-related factors (e.g. content, location, participant involvement in development and implementation, possibility to maintain social contacts) [11, 15, 17–19]. In addition to social strata and sex (gender), several individual-level factors also play a role (e.g. age, health status, attitudes, knowledge, needs, motives, perception and type of activity) [9, 13–15, 20, 21].

When analysing reasons for non-participation at the individual level, it is important to distinguish between a lack of awareness or misconception regarding a PA offer, and active decision-making. While some older adults are not aware of, or might not understand the concept of PA programmes, others make a conscious decision not to participate in PA interventions. Reasons for non-participation include time pressure, lack of interest and knowledge, health problems, fear of not being able to keep up, being already sufficiently active, or not believing that PA will have a positive long-term impact on one’s health [11, 20, 22, 23]. Although older adults more often refer to health benefits as a reason for PA compared to younger adults [24], proximal outcomes (e.g. positive emotions) have also been found to be of importance for them [25, 26]. Studies among non-participants have also shown that the specific content and design of an intervention might hinder participation [13, 23]. Recruitment strategies for PA interventions often include public health messages about PA that are developed under the assumption that health benefits are important enough for the target group to participate [27]. However, such messages do not appeal to all older adults in the same way [11, 15, 21]. Consequently, messages used for recruitment greatly influence participation in PA interventions focusing on behavioural change [22]. There is little evidence for the effectiveness of message tailoring in health promotion interventions and so far, only a few studies addressing PA in (older) adults have tailored health information to different levels of attitudes, motives and knowledge, or target groups [28].

Although available research has already identified several factors that affect older adults’ participation in PA programmes, everyday life processes and orientations that drive the decision to participate in a PA programme are not yet well understood. Analysing reasons for (non-)participation from an everyday life perspective provides the potential to gain a deeper understanding of the target group. This, in turn, may inspire PA interventions to accommodate different orientations and motivations. The aim of this study was thus to identify factors associated with participation in a PA intervention from an everyday life perspective, and to construct a typology that reveals patterns of PA participation behaviour of older adults.

## Methods

This qualitative study was part of the Ready to Change project [6], which was part of the first phase of the AEQUIPA prevention research network (Physical activity and health equity: primary prevention for healthy ageing) [29]. Ethical approval for this study was obtained from the Ethics Commission of the University of Bremen, Germany.

### The PA intervention

The starting point of this qualitative investigation was the ‘Fit in the Northwest’ intervention study, in which the effectiveness of two web-based interventions to promote PA in older adults was investigated [7, 30]. The intervention consisted of a 10-week programme including web-based activity diaries, fitness trackers, brochures with recommendations for PA and a weekly group meeting, during which practical exercises and information on healthy lifestyles were offered [8].

### Sample and recruitment

Individuals were eligible for participation in the qualitative interviews if they were 65 to 79 years old and had either participated or consciously decided against participating in the PA intervention. Although we were primarily interested in the perceptions and reasons given by those who had not participated in the programme, we also included programme participants in the interview study as a control group. Those who had decided not to take part in the PA intervention due to health-related reasons were excluded from the sample. The recruitment of non-participants was carried out using a combined procedure comprising direct (pick-up on site, information flyers) and indirect strategies (gatekeeper, written invitation to non-participants registered in study-database).

### Interview guide development

For an in-depth analysis of the reasons for or against participation, we set up an interview guide for episodic interviews. In episodic interviews, narrative-episodic knowledge (narratives) is linked with semantic knowledge (concrete knowledge on targeted questions) [31]. Thus, this form of interview represents a combination of methods and opens up multi-perspective insights. First, possible topical areas were identified using Anderson’s behavioural model of health service use [32] and Ryan and Deci’s self-determination theory [33]. Guiding questions and probes on different topical areas were then formulated. Ryan and Deci’s theory was used as the background for the areas of PA biography, user experiences and health-related attitudes and health behaviour, while questions about the specific PA intervention and needs with regard to PA were guided by the Anderson model (see Additional file 1: Table S1). The interview guide was piloted with two older adults of different sexes and accordingly adapted in terms of comprehensibility and content. Social-demographic information and PA level of the interviewees were collected using a questionnaire.

### Interview process

Based on the recommended number of 20–30 cases needed for achieving data saturation in episodic interviews [34], 25 episodic interviews were carried out from March to May 2017. Most of the interviews were conducted at the interviewees’ home and the rest at the interviewer’s office or a public institution close to the interviewee’s place of residence. At the beginning of each interview, the participants gave their written consent to participate in the interview. The average length of interviews was 60 min (range 30–120 min). All interviews were audio-recorded and transcribed verbatim using the F4 software. The transcripts were then analysed by developing an inductive coding system using MAXQDA (version 10). Sections that are relevant for this manuscript as well as the interview guide were translated into English (see Additional file 1: Table S1).

### Data analysis

The interviews were analysed according to Corbin and Strauss’ version of the Grounded Theory methodology [35]. In line with the principle of the greatest possible openness, the dimensions and categories in the analysis were developed from the empirical material. During this process, the developing dimensions and categories were constantly compared, modified and verified [31, 34, 35].

The construction of the typology of PA participation behaviour comprised four steps [36]: (1) Selecting the dimensions which proved to be explanatory regarding the reasons for the decision for or against participation; (2) Grouping of the cases according to their position on the different analytical dimensions supported by interview quotes; (3) Analysis of content-related meanings; (4) Characterization of the identified types of PA participation behaviour. In cases where an interview contained aspects of different types, a comparative weighting of the statements was done.

In order to increase data credibility, reliability, reflexivity as well as relativity and to reduce the risk of bias, peer debriefing strategies (regular communication with other qualitative researchers) were implemented [37, 38]. The results of individual analysis steps of the Grounded Theory (Free Coding, Axial Coding and Selective Coding) were elaborated and further developed by two scientists at regular intervals. Important milestones of data analysis (key categories, central phenomenon and final typing) were validated in three qualitative research workshops attended by four qualitatively researching scientists. For instance, selected sections of the transcripts were (re)-coded independently and then reflected upon and discussed together. All deviations were discussed in the plenum until a consensus was reached.

## Results

### Characteristics of the interviewees

A total of 25 people were included according to a pre-defined 2:1 ratio of non-participants and participants. Twenty-two of the interviewees were recruited through direct recruitment procedures (written invitations, personal contact) and three through indirect procedures (local gatekeeper). The interviewees (17 non- participants and 8 participants) were 71.5 years old on average and most of them were women (15/25). All interviewees, apart from two, originated from Germany and more than half lived alone in their own household. Only two of the interviewees rated their health status as being poor. Further, three non-participants and four participants stated that they were physically active less than 4 days a week (see Table 1).

**Table 1:** Characteristics of interviewees

	Non- participants (*n* = 17)	Participants (*n* = 8)	Total (*N* = 25)
Age in years (SD; range)	71.9 (3.97; 66–79)	70.5 (2.88; 68–77)	71.4(3.65; 66–79)
	n (%)	n (%)	N (%)
Sex			
Men	7 (41.2)	3 (37.5)	10 (40.0)
Women	10 (58.8)	5 (62.5)	15 (60.0)
Country of birth			
Germany	15 (88.2)	8 (100.0)	23 (92.0)
Other Country	2 (11.8)	–	2 (8.0)
Education			
Secondary school or less	10 (58.8)	7 (87.5)	17 (68.0)
Higher education	6 (35.3)	1 (12.5)	7 (28.0)
Other	1 (5.9)	–	1 (4.0)
Residential area			
Urban environment	17 (100.0)	6 (75.0)	23 (92.0)
Semi-rural environment	–	2 (25.0)	2 (8.0)
Persons in the household			
1 person	8 (47.1)	4 (50.0)	12 (48.0)
2 persons	9 (52.9)	4 (50.0)	13 (52.0)
Self-rated health^a^			
Good	15 (88.2)	8 (100.0)	23 (92.0)
Poor	2 (11.8)	–	2 (8.0)
Minimum 30 min moderate to vigorous PA at least 4 days / week			
Yes	14 (77.7)	4 (22.2)	18 (72.0)
No	3 (42.9)	4 (57.1)	7 (28.0)

^a^ Five response options (excellent, very good, good, fair, poor) dichotomised into good (excellent, very good, good) and poor (fair, poor)

### Thematic analysis results

The interview results are presented as components of a dynamic model of physical activity participation behaviour reflecting the perspectives of the interviewees. The different types of participation behaviour resulting from the different key factor perspectives are clarified in the following section. Thereafter, additional factors that could have had an influence on the typification of participation behaviour are presented. In the final section, results on participation in the intervention according to types of PA participation behaviour and the characteristics of the interviewees are presented. Exemplar quotes are included in the main text, with additional supporting quotes presented in Table 2.

**Table 2:** Results of the interviews – Examples of quotes^a^

Theme	Number	Quote
Dynamic model of the participation behaviour		
	Q1.1	*“My husband has been retired for 2 years. He worked until he was 65 years old and we have time, we are managing our own time and we will try to enjoy our lives to the most.” [VNT004,49,non-participant]* *“It’s like, I try to enjoy each day, you can never know how quickly it may be over.” [VNT007:78,non-participant]*
	Q1.2	*“We both volunteer, so that, too, is one of the positive things.” [VNT008:51,non-participant]*
	Q1.3	*“It’s like this: we would like to get rid of the obligations we had throughout or lives and which we enjoyed fulfilling, that’s not the question, I enjoyed raising children.” [BNT007:58]*
	Q1.4	*“Regarding the process or whether one regards a certain sport as being sensible and so on, you pick what suits your type and what you still have the strength for. For example, I cannot play tennis anymore” [VNT010:129, non-participant]*
Key factors related to perceived fit of the PA intervention		
Time management of everyday life	Q2.1.	*“Usually, in summer, I am on the golf course two or three times a week. And my additional exercise has been going to the gym on a regular basis for 8 or 9 years.” [VT001:8,participant]* *“So, I exercise two or three times a week, plus I go hiking once a month, I would do that twice, though [ … ]. I need to have fixed appointments, at least partly.” [VNT009:62,71,non-participant]* *“But if I do participate it means that there will be a meeting once a week and this will be scheduled. It is not optional.” [BT001:55,participant]*
	Q2.2	*“I no longer make plans other than on the spur of the moment. I can call the travel agency tomorrow and ask whether they have anything. And if they do I will be at another place the day after. I am living spontaneously and enjoying the present.” [VNT005:85,non-participant]* *“I always have the feeling that I do not like to be tied, I think. Don’t know if I am right, just my thoughts. I want to do what I want at any given hour. Thus, I don’t like to be told what to do.” [BNT007:38,non-participant]* *“I am, I hadn’t heard of it before and basically I wouldn’t have minded to participate. But that would have meant being tied in terms of time and I did not want that because we have been travelling a lot.” [VNT008:63,non-participant]*
	Q2.3	*“We have all been in this sports club for 40 years [ … ]. All seniors, just 14, 15, 16 people. And we go there every Tuesday. Gymnastics and volleyball, in former times [ … ].” [BNT005:11,non-participant]* *“I have been there since I was young and I still do their accounting, too. One, twice, three times a month.” [BNT005:33,non-participant]*
	Q2.4	*“If I do something like this there is indeed some group pressure, but a very relaxed one. [ … ] That goes for any type of sport I would engage in.” [VNT006:154,non-participant]*
Goals addressed primarily with PA	Q2.5	*“I have this pedometer and so I always have a clue. Today I did 4.000 steps and the day before that I did 15.000 steps. So I manage this and will do so, eventually, regarding food.” [VT002:14,participant]*
	Q2.6	*“I am individualistic and wish to exercise accordingly. Hiking is okay, no doubt. Enjoying and feasting from the landscape is nice but please no high performance. If I have to be competitive I lose all desire.” [VNT009:25,non-participant]* *“A walk there and back, on the beach. It is so expansive, you don’t even notice the 10 kilometers. Only the vastness of the landscape.” [VNT001:185,non-participant]*
	Q2.7	*“As for me, I consider it sensible, physical activity as well as establishing social contacts.” [VT001:12,participant]*
	Q2.8	*“For me it’s good that I can rate myself. I find this very important for me. And afterwards I pat myself on the shoulder and say ‘It’s not that bad yet.” [VT002:62,participant]* *“When I am physically active and I feel that my body is still cooperating I think that’s positive. I get the feeling that it’s good being able to do today what many younger persons cannot. I have to say that I am sometimes very proud when I look around myself.” [OT001:24,participant]*
Multidimensional typology of participation behaviour		
‘Health designer’	Q3.1	*“[ … ] that it would be obvious for me to attend a meeting of the program, there is a reason for it.” [BT001:49,participant]*
	Q3.2	*“[ … ] and in case you have a day which is not so good, well, you have to overcome your weaker self.” [VNT007:74,non-participant].*
	Q3.3	*“[ … ] initially, I liked the idea very much to do this under supervision. Basically, I liked it but as I said before, it simply became too much for me. I had joined a sports club, I wanted to play golf again, I wanted to pursue my other leisure activities.” [VNT004:51,non-participant]* *“Usually, in the summertime I am on the golf course for two or three times a week. Plus the exercise I get by going to the gym on a regular basis for 8 or 9 years now [ … ]. Adding to that, I spend about 45 min on the ergometer, biking steadily because riding my bike into town is not so great for the heart.” [VT001:8,participant]*
	Q3.4	*“Since I retired, I usually always carry a pedometer in my pocket so that I can check whether I have walked enough in the evening.” [VNT004:9, non-participant]*
‘Flexible Function-oriented type’	Q3.5	*“I don’t consume alcoholic beverages and I don’t smoke. Even my doctor said “Well done, good decision.” [VNT006:76,non-participant]*
	Q3.6	*“Particularly when you get older, it is important to stay fit [ … ]. I can feel it now that it won’t be possible without physical activity. But is has to be done at a level that one can decide for themselves and spontaneously, whatever and wherever.” [VNT006:12,126,non-participant]* *“That I am not tied, it has to be an open and casual group in which I can decide for myself.” [VNT001:527, non-participant]*
‘Comparison and competition type’	Q3.7	*“When you are successful through sports and physical activity you almost automatically have a nice evening.” [BNT001:90,non-participant]*
	Q3.8	*“And then I thought I am joining in, just to find out whether in a comparative group or amongst the people who also joined in, well, whether I am the weakest one who always hobbles behind or whether I am average [...].” [VT002:32,participant]* *“[ … ] and whenever new and young men join the group and fail I say to myself it’s good you joined and stayed.” [OT001:24–28,participant]*
‘Fun and Wellness-oriented type’	Q3.9	*“I only exercise when I enjoy it. But I don’t consider that as health and fitness training.” [BNT007:8,non-participant]*
	Q3.10	*“I am individualistic and wish to exercise accordingly. Hiking is okay, no doubt. Enjoying and feasting from the landscape is nice but please no high performance. If I have to be competitive, I lose all desire.” [VNT009:25,non-participant]* *“I enjoy geocaching like you wouldn’t believe it. There are the most awesome hiding places [ … ]. Yes, I very much enjoy that.” [BNT004:76,non-participant]*
Dynamic factors influencing the type of participation behaviour		
Biographical experience with structured PA programs	Q4.1	*“In winter, as a pupil I participated in the sledding championships with my friend and we ended up first. We received a certificate.” [BT001,23,participant]*
	Q4.2	*“Boys like to define themselves by physical performance and in that regard I was always at the back. That frustrated me somewhat so that I gave it up then.” [VNT008:45,non-participant]*
	Q4.3	*“Yeah, gym class! I’ve always enjoyed that, actually. I was always there, too.” (OT001:14,participant)*
	Q4.4	*“Actually, it was a compulsory subject. You didn’t have a choice. You had to do sports and be physically active.” [VNT001:5,non-participant]*
Awareness of physicals signs of ageing	Q4.5	*“You aren’t getting younger. Some things don’t work out the way they used to, but I think one has to take a bit of care of the things one is still capable of doing.” [VT001:8,participant]*
	Q4.6	*“I know from experience that time and again I will come to a point where I do too much for myself, then I hang around and develop bad feelings.” [VNT002:58,non-participant]*
Social integration	Q4.7	*“If one lives alone it might happen that some days you think to yourself ‘Oh my god, I haven’t spoken to anyone today,’ if one doesn’t go out.” [VNT007:54,non-participant]*
	Q4.8	*“Then take care of grandchildren a little more, meet our friends, relieve the children but first of all we want to travel. [ … ] This obligation to go someplace. That’s it, the group pressure and fixed appointments which is what I’ve had for so many years.” [VNT006:96,126,non-participant]*

^a^To improve readability the quotations have been linguistically polished to a small extent

### Dynamic model of physical activity participation behaviour

The interviewees’ responses reflected the fact that they were in the “older age” phase of life [Q1.1], with participation decisions being made based on past experiences and increased time availability. Consequently, the use of time and related attitudes towards commitments emerged as a major theme among the respondents. They showed different attitudes towards life-phase specific obligations (e.g. honorary posts, responsibility for grandchildren, as well as sports clubs and PA programmes with fixed structures and contents), which seemed to be influenced by their past experiences (work, family, leisure). For example, while some respondents found commitments useful and positive [Q1.2], others found them to be time consuming and restrictive, and wanted to have less of them [Q1.3].

The content and structure of the PA intervention proved to be of critical importance for the decision whether or not to participate: *“Regarding the process or whether one regards a certain sport as being sensible and so on, you pick what suits your type and what you still have the strength for. For example, I cannot play tennis anymore.”* [VNT010:129, non-participant] [Q1.4]. The interviewees assessed the suitability of the PA intervention in relation to both individual conditions and their social context. They referred to two key factors: the perceived benefits related to their individual PA goals, and the time management preference in everyday life.

#### Goals achieved primarily through PA

The goals that motivated PA differed between the interviewees. In principle, all respondents identified an increase in physical and mental well-being as a major benefit of PA. However, a more detailed analysis revealed different poles ranging from the extremes ‘health control’ to ‘sense of indulgence’.

For ‘health controllers’, long-term well-being played a central role. This they aimed to achieve by consciously controlling body functions that, among other things, influence and signal physical fitness, mobility and general health [Q2.1]. Those at the pole of ‘sense of indulgence’ placed high relevance on proximal well-being, such as fun, joy and satisfaction resulting directly from being physically active [Q2.2]. Two further positions evolved between the poles ‘health control’ and ‘sense of indulgence’, namely, ‘socializing for health’ and ‘social comparison’. In contrast to the ‘health control’ group, those belonging to the pole ‘socializing for health’ linked long-term health-related goals and social contacts with PA [Q2.3]. The ‘social comparison’ group on the other hand primarily aimed to achieve a sense of well-being through comparing their PA performance to that of their peers [Q2.4]. Regarding primary activity goals, ‘socializing for health’ group members were closer to the pole of ‘health control’, and the ‘social comparison’ members to the pole ‘sense of indulgence’.

#### Time management of everyday life

The interview material revealed large differences in how the respondents managed their available time in everyday life. The structuring of time ranged from complex arrangements of diverse, scheduled or organised activities in different places not close to where they live [Q2.5], to a minimum of scheduled obligations and high relevance for spontaneous activities [Q2.6]. Between these poles, we identified further positions regarding the degree of time structuring. Some respondents showed a local structuring of time, which was more oriented towards activities organised close to home [Q2.7]. Others preferred activities with limited time commitments [Q2.8]. The preferences of the time management of positions ‘complex structuring’ and ‘local structuring’ were classified as structured time management in everyday life, whereas the positions ‘limited time commitments’ and ‘flexible individualism’ were regarded as flexible time management.

#### Multidimensional typology of PA participation behaviour

**Fig. 1:**
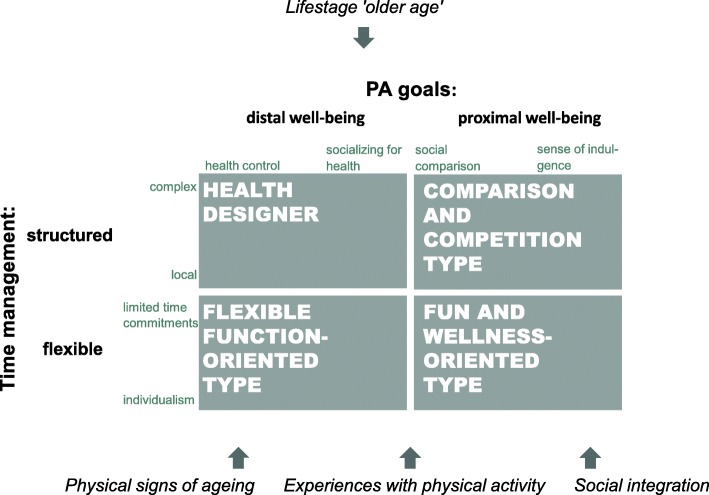
Typology of physical activity participation behaviour based on the perspective of older non-participants and participants of a PA intervention, incorporated in the dynamic model

The multidimensional typology of PA participation behaviour combines time management preferences and PA goals, resulting in four types of PA participation behaviour (see Fig. 1).

##### Health designer

This type was characterised by an orientation towards fixed time structures; various organised offers were taken up at regular intervals as a matter of course, and participation in organised PA activities was perceived as an integral part of everyday life. The main goal was long-term health maintenance, with a focus on certified courses provided by institutions and established organisations, such as insurance-funded exercise programmes [Q3.1]. Interviewees with this type of participation behaviour had a high level of health literacy and saw themselves as experts of their own health. They were also willing to accept short-term negative effects (e.g. pain) [Q3.2] in order to achieve their long-term goal. To this end, organised formats such as the certified courses mentioned afore were preferred [Q3.3] and various strategies such as technical aids (e.g. apps and pedometers) were used as health checks: *“Since I retired, I usually always carry a pedometer in my pocket so that I can check whether I have walked enough in the evening.”* [VNT004:9, non-participant] [Q3.4].

##### Flexible function-oriented type

Similar to the PA participation behaviour ‘Health designer’, the ‘Flexible function-oriented’ type was characterised by a PA behaviour oriented towards goals to address long-term well-being as well as established institutions or professional experts (health insurances or doctors) [Q3.5]. With regards to performing PA, great importance was given to general health, maintaining physical fitness and mobility. In contrast to the ‘health designer’, this type wavered between the desire for PA programmes that promised sustainable health effects and the wish for flexibility and room for spontaneous activities. Those belonging to this participant behaviour type did not want social and other obligations to take up a lot of their time. They hence preferred individualised PA formats in the form of individualised PA programmes in fitness centres or open sports groups with a low participation requirement: *“Particularly when you get older, it is important to stay fit [ … ]. I can feel it now that it won’t be possible without physical activity. But is has to be done at a level that one can decide for themselves and spontaneously, whatever and wherever.”* [VNT006:12,126, non-participant] [Q3.6].

##### Comparison and competition type

In contrast to ‘Health designer’ and ‘Flexible function-oriented type’, the primary aim for this type was proximal well-being attained during or immediately after participating in PA. This type was characterised by an orientation towards social comparison and the respective participants were motivated by the recognition of their physical performance. This, according to the respondents, was achieved through positive feedback from others [Q3.7] as well as by comparing their own performance to that of peers or persons of a younger age group. This type wanted to show that people of retirement age were not old scraps: *“And then I thought I am joining in, just to find out whether in a comparative group or amongst the people who also joined in, well, whether I am the weakest one who always hobbles behind or whether I am average [...].”* [VT002:32, participant] [Q3.8].

##### Fun and wellness-oriented type

The PA participation behaviour of this type was primarily guided by proximal positive emotions directly related to exercising. Having fun, well-being and joy were factors that motivated those belonging to this type to exercise PA. The type lived more in the ‘here and now’ and enjoyed life after retirement. Similar to the ‘Flexible function-oriented type’, there was a pronounced need for autonomy with regard to the management of PA in leisure time. The goal was to minimise obligations and achieve a high degree of individualism. Ageing or physical limitations that could have a negative impact on future health and physical performance only had little impact on current participation behaviour [Q3.9]. In contrast to the ‘Comparison and competition type’, the ‘Fun and wellness-oriented type’ disliked the idea of competition, rather striving to experience indulgence and fun through various PA offers or their surroundings. These activities were potentially adapted, for example, according to the weather or how the respondents felt at a particular moment in time: *“I am individualistic and wish to exercise accordingly. Hiking is okay, no doubt. Enjoying and feasting from the landscape is nice but please no high performance. If I have to be competitive, I lose all desire.”* [VNT009:25, non-participant] [Q3.10].

### Additional factors influencing the type of participation behaviour

The statements of the interviewed (non-)participants indicate that additional factors such as biographical experience with structured PA programmes, perception of physical signs of ageing and the degree of social integration had a dynamic influence on the types of participation behaviour.

#### Biographical experience with structured PA

The interviewees had very different experiences with PA and had been exposed to different formats of PA over the course of their lives. While some reported positive key experiences during school sports or from sports club activities in childhood and adulthood [Q4.1], others had rather negative key-memories [Q4.2]. The respondents also perceived participation in school and club sports differently. Whereas PA meant social recognition and fun for some [Q4.3], others felt that participation was only a social obligation [Q4.4]. Positive experiences tended to be more associated with participation behaviour types ‘Health designer’, ‘Comparison and competition’ and ‘Fun and wellness-oriented’, and negative experiences with ‘Flexible function-oriented type’.

#### Awareness of physicals signs of ageing

The interviewees were confronted with varying degrees of physical signs of ageing. The personal physical condition and resilience in relation to PA were perceived differently by the interviewees. While noticeable physical deficits were seen as a reason for taking up a health-related active lifestyle by some [Q4.5], others were more afraid of not being able to keep up or of worsening the problems through sport and PA [Q4.6]. Although signs of ageing were interpreted differently, the perception of signs of ageing as a reason to take up a health-related active lifestyle was more frequent among those whose participation behaviour was oriented towards long-term health, that is, ‘Health designer’ and ‘Flexible function-oriented type’.

#### Social integration

The interviewees had different degrees of social integration. Some reported that they lived alone after the death of a partner and that activities outside their own home offered the possibility of communication and structuring of their everyday life [Q4.7]. Others on the other hand reported having a large circle of friends with whom they planned many regular leisure activities (e.g. PA). A number of respondents living in a partnership regularly took care of their grandchildren and hence preferred flexibility in PA [Q4.8]. A perceived lower degree of social integration of the interviewees was more frequently associated with the more structured type ‘Health designer’ and ‘Comparison and competition’.

### Types of PA participation behaviour, characteristics of interviewees and participation in the PA intervention

**Fig. 2:**
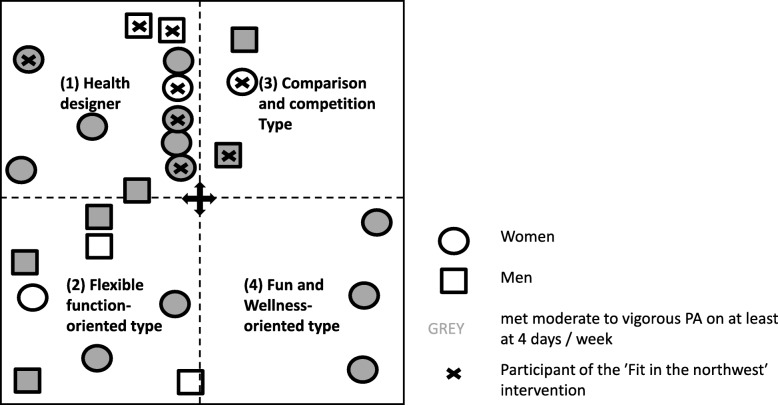
Participation in the PA intervention according to the type of physical activity participation behaviour and the characteristics of the interviewees

The majority of the 11 interviewees assigned to the type ‘Health designer’ were women (*n* = 8). In contrast, there were more men than women among the ‘Flexible function-oriented type’ (5 out of 8). In addition, all rather inactive non-participants were assigned to the ‘Flexible function-oriented type’. Interviewees who had participated in the PA intervention all belonged to the ‘Health designer’ and the ‘Comparison and competition type’ (see Fig. 2).

## Discussion

The results of this work complement the findings from previous research by integrating different factors influencing participation in PA intervention in older age into a multidimensional typology.

When deciding whether to participate in the PA intervention or not, the central question for the participants in our study was if the intervention was suitable for them. The answer to this question was grounded in their individual goals in relation to PA and their leisure time management. Based on these two factors, four different types of PA participation behaviour emerged. Similar to previous findings [24], distal PA goals, in the form of health benefits, were the main reason for being physically active given by the older adults in our study. The differences in behaviour types, reported individual attitudes and motives for PA as well the preference for specific temporal formats observed in our study have also been observed in other studies [9, 11, 13, 17, 20].

The health related goals of the first two PA participation behaviour types, ‘Health designer’ and the ‘Flexible function-oriented’, are in line with the results of a review of older people’s reasons for participating in PA [15]. The main goals of the interviewees classified as belonging to these two types were their long-term health and well-being, which is in line with the findings reported in the majority of the studies included in the said review. The ‘Comparison and competition’ and ‘Fun and wellness oriented’ types on the other hand, had more proximal goals whose relevance in terms of motivation and expectations for participation in PA should not be underestimated [25–27].

In accordance with the self-determination theory [33], our results show that both intrinsic motives (‘Health designer’, ‘Flexible function-oriented type’, ‘Fun and wellness oriented type’) and extrinsic motives (‘Comparison and competition type’) played a role in determining the participation behaviour. Other studies on motives for participation show similar tendencies and a consistent positive association between intrinsically oriented motives and participation in PA [39]. Only a few statements relating to long-term health-oriented well-being, which could be regarded as both intrinsic and extrinsic motives were reported in this study (e.g. activity recommendation by the physician). While a doctor’s recommendation is obviously an external factor, taking care of one’s health – the underlying goal of following the doctor’s advice – is by virtue an intrinsic factor [39].

Regarding time management of PA, although previous results did not typify PA participation behaviour the way we did, there are similarities between our findings and those reported in the literature [22, 23]. As observed in our study, the lack of leisure time and problems with the duration and structure of PA were cited as reasons for non-participation. The temporal preferences observed in our study also provide valuable information on how older people currently deal with obligations. The nature, extent and management of post-employment obligations are reassessed on an individual basis, with some older people minimising obligations and others organising newly created leisure time through obligations (e.g. volunteering, PA and sports) [40].

In accordance with the paradigm of grounded theory, we observed additional factors such as biographical experiences, perception of physical signs of ageing, and social integration, that affected the types of PA participation behaviour identified. A change in one or more of these factors may result in a dynamic process involving the (re-)assignment to a different type of PA participation behaviour [35], thereby posing a challenge for interventions.

The persons interviewed in our study reported both positive and negative previous experiences with structured PA, made in the context of school sports, club sports, or rehabilitation. While the reported experiences could not be clearly identified as being either beneficial or hindering factors for the perceived suitability of the PA intervention, it could nevertheless be shown that the experiences from childhood and early adulthood were very present for many interviewees. Further, it was evident how these experiences can influence the type of participation behaviour, even at older age. Those reporting positive experiences, such as fun and success in school sports, also tended to positively evaluate and prefer structured PA programmes more often than those reporting negative experiences. This aspect of experiences with structured sport has hardly been focused on by previous studies. The incorporation of life course-related approaches in future studies could help bring more clarity on this.

The perception of physical signs of ageing was also identified as an additional factor in the model of participation behaviour in our study. Age-related physical deficits were seen as both a reason for the increase in PA as well as an inhibiting factor, and were associated with the types that address long-term well-being with PA. This finding is in contrast to previous qualitative studies in which physical limitations, lack of fitness or health deficits were only cited as barriers for participation in PA [11, 17, 20, 22, 23].

The statements of the interviewed older adults reflect different degrees of social integration. Accordingly, some non-participants reported involvement with high social commitments and many competing events. While a high degree of companionship and social support has been identified as a beneficial factor in previous research [17], among our interviewees a high degree of social contacts and commitments seemed to be accompanied by a lack of time and not necessarily perceived as being contradicting the beneficial aspect for participation.

### Suitability of PA intervention to needs of different behaviour types

In the case of PA intervention, only the type of ‘Health designer’ and ‘Comparison and competition type’ seemed to have a high degree of fit. The interviews indicate that some respondents perceived the time structure and recurring appointments of the PA intervention as constraining and restrictive. These reasons correspond to the PA participation behaviour of two identified types: ‘Flexible function-oriented’ and ‘Fun- and wellness-oriented’. The typical characteristics of these types suggest that, in relation to the offered PA intervention, a high degree of fit with the format and/or objectives was not achieved. For example, the results indicate that more inactive men are more likely to respond to flexible health-oriented PA programmes rather than to the structured formats we offered.

### Strengths and limitations

The strength of this qualitative approach is that it does not focus on isolated aspects of the participation behaviour of the group of non-participants. Instead, it follows the assumption that older non-participants and participants have different attitudes, experiences and lifestyles that explain their participation behaviour. Hence, a first attempt was made to construct a typology of PA participation behaviour based on the everyday perspective of older non-participants and participants of a community based PA intervention. The typology does not only describe one-dimensional superficial phenomena and phenomena of the context, but rather provides a possible multidimensional explanation.

Recruiting persons who decided not to take part in an intervention study for an interview is very challenging. In order to obtain the greatest possible variance in the sample, different recruitment methods were applied. Nevertheless, for practical research reasons (time limitation), we were not able to carry out theoretical sampling according to Grounded Theory methodology [35], so that a targeted case selection was no longer possible during the data analysis. However, the analysis of the three interviews conducted last did not reveal any new descriptive codes or topics, so that data saturation can be assumed for this sample.

Further research should examine whether the attitudes and lifestyles of specific sub-groups (e.g. very inactive persons, persons with a migration background) include other types. The results of this explorative work should however be interpreted with caution as the behaviour types presented were constructed based on the experiences and views of a small number of respondents and cannot be taken to be representative for the older population aged 65 and over. It should also be borne in mind that the types of PA participation behaviour constructed in this study are ideal types which are not found in this form in social reality. In reality a person can have characteristics of different behaviour types, which can change depending on various reasons such as sense of well-being at a particular point in time or other life events. Including structural aspects such as the type of provider of PA, the place of the intervention and distance from home in the analysis, factors which are also known to influence the success of PA research and practice [13], might have enhanced the results. The focus of the study was however on individual factors. Discussing the assignment of the typologies to the interviewees with the respondents would have contributed to the quality of the results. This unfortunately could not be carried out due to data protection regulations.

### Practical implications

Despite the limitations, our findings have several practical implications regarding recruitment strategies and formats or the design of studies for the promotion of PA for older people.

In particular, the reach of PA interventions in older age groups can be strengthened, e.g. through more tailored communication in recruitment. In addition to structural factors, personal factors such as different goals, preferences and lifestyles of the target group should be incorporated. Our typology of PA participation behaviour suggests that:

- PA interventions should address both proximal and long-term goals for PA
- Ideally, PA interventions should include both flexible and structured components and allow for choice

The aim of this study was not only to optimise the planning and design of PA interventions with regard to the subjective perspectives of a heterogeneous group of older adults of retirement age, but to also improve specific implementation activities such as the design of target group communication. In general, the information and messages of different recruitment strategies (e.g. invitation flyers, postal invitations) are developed by practitioners or researchers under the assumption that health is of high value or an important goal for the members of a target group. Often, the messages or images used are aimed exclusively at classic formats of a long-term healthy lifestyle. Our results show that the development and use of such messages and/or materials need to take the different PA goals (e.g. positive emotions) and needs for choice in relation to the design of PA programmes more into account. Message framing and message tailoring [41] are one way of systematically addressing the different types of PA participation behaviour in recruitment and implementation process.

## Conclusions

In this study, the expectations of older adults with regard to PA interventions could be better understood due to the subject-related analysis approach used. The identified types of PA participation behaviour can serve as valuable supplementary information to that on age, gender and level of PA characteristics during the design, recruitment and implementation of PA interventions for specific target groups. However, the planning, development and implementation of interventions of primary prevention cannot follow a one-sided or highly specific orientation. In other words, it is not possible to offer every older person an individual PA intervention. Nevertheless, subjective reasons for participation behaviour should be known in order to increase the take-up and effectiveness of PA interventions among the elderly.

## Supplementary information

**Additional file 1: Table S1.** Interview Guide: Reasons for (non-)participation in a structured PA intervention.

## Data Availability

The datasets analysed during the current study are not publicly available because the data collection as approved by the ethic committee did not allow for making them publically available.

## Abbreviations

AEQUIPA: Physical activity and health equity: primary prevention for healthy ageing
PA: Physical activity
WHO: World Health Organization
