# Supplementary material for: Ready to participate? Using qualitative data to typify older adults’ reasons for (non-) participation in a physical activity promotion intervention
Source: BMC Public Health. 2019 Oct 22;19:1327. doi: 10.1186/s12889-019-7688-y (PMC6805576; doi:10.1186/s12889-019-7688-y)
Supplement: Supplementary file 1 — Additional file 1: Table S1. Interview Guide: Reasons for (non-)participation in a structured PA intervention. [file 12889_2019_7688_MOESM1_ESM.docx]

| **Additional file 1: Table S1** Interview Guide: Reasons for (non-)participation in a structured PA intervention | |
| --- | --- |
| **1. Biographical experiences associated with exercise or offers for physical activity from third parties/ „activity“ biography/ user’s experience** | |
| To begin with, I would like to ask you to tell me what importance physical activity and exercise have in your life so far. Starting from youth, please recount to this date. | |
| Questions:   - During which stages of your life were been particularly active compared to others. What did this depend on? - What types of activities did you prefer? How did you come about to choose these activities? (Systemization of sports: team sport, individual sport ….) - What attitude do you think most of your friends and acquaintances have towards physical activity? - Which positive and negative experiences do you associate with physical activity/ exercise? | Probes:   - Please tell me from your childhood (pre-school), school times, early adolescence, adulthood, today. (Enquire about the different stages if necessary). - What else was important in connection with physical activity? - What do friends and acquaintances think about exercise? Or: - How important is or was your friends’ attitude towards physical activity still for you? - What kind of attitude do your friends have towards physical activity? - Do you recall particularly positive or negative experiences from your childhood or adolescence? |
| **2. Autonomous life goals** | |
| Please describe a day which felt particularly nice or successful: What did you do, where and with whom? | |
| Questions:   - You are now between 60 and 75 years old. How do you wish to live your life in the next years? What plans and activities are important to you? | Probes:   - Even though you may not be able to accomplish everything in one day, what further experiences and activities would you include in a successful day? - What further plans and activities can you think of? - Are there any activities which are of particular importance to you? |
| **3. Assessing offers for activity** | |
| What were your thoughts upon first hearing/ reading about ′Fit in the Northwest′? What part of the offer appealed to you? What did not? What was decisive for your choice? | |
| Questions for participants:   - What personal benefit do you expect from participating in this offer? - What is your assessment regarding the location, accessibility, expenses, scheduling, awareness level? - What is your assessment of the provider and local connectivity?   Questions for non-participants:   - In your opinion, what kinds of people will participate? - What do you think about possible trainer of this offer? | Probes   - What else was on your mind? - What idea did you have/ do you still have about this offer when you first read or heard about it? - What else did you consider positive? - What else bothered you? - What further personal benefit do you see? - Are there further criteria which affected your decision? |
| **4. Wishes/ hypothetical question regarding activity offers** | |
| Presuming, you had been granted a wish for a whole new tomorrow with an activity offer you would be happy to participate in. What would that offer have to look like? | |
| Questions:   - In what way would an activity offer have to be organized for you to see a benefit in it? - In what way would it have to be organized regarding accessibility, expenses, scheduling, local connectivity? - Who besides yourself would have to participate?   Who should supervise this offer? | Probes:  What further features would this offer have to have? |
| **5. Closing Statement** |  |
| Thank you very much. These were all my questions. Is there anything else concerning this subject you would like to add? | |
